# Preparation of cholesterol-imprinted polymer for selective adsorption of cholesterol from gastrointestinal mimicking solution

**DOI:** 10.55730/1300-0527.3666

**Published:** 2024-02-07

**Authors:** Veyis KARAKOÇ, Erol ERÇAĞ

**Affiliations:** 1 Vocational School of Health Services, Çankırı Karatekin University, Çankırı, Turkiye; 2 Department of Chemistry, Faculty of Engineering, İstanbul University-Cerrahpaşa, İstanbul, Turkiye

**Keywords:** Molecularly imprinted polymers (MIP), intestinal mimicking solution (IMS), cholesterol removal, pseudospecific ligands, methacryloamidotyrosine (MATyr)

## Abstract

The purpose of this study is to synthesize a highly selective adsorbent to remove cholesterol, one of the most important causes of cardiovascular diseases, from the intestinal mimic solution (IMS). For this purpose, cholesterol imprinted polymers were synthesized by suspension polymerization method using the molecular imprinting technique. In the first step, the functional monomer MATyr with hydrophobic character was synthesized. Then, the cholesterol-MATyr monomer precomplex was formed and the polymerization process was carried out by adding cross-linkers with the comonomer HEMA. The synthesized polymer poly(2-hydroxyethyl methacrylate-N-methacryloyl-(L)-tyrosine methylester) poly(HEMA–MATyr) was characterized by FTIR and SEM. The cholesterol adsorbing behavior of the synthesized poly(HEMA–MATyr) microbeads adsorbent was investigated at different initial concentrations, different temperatures, and adsorption times. The maximum adsorption capacity of microbeads was determined as 56.67 mg/g at a concentration of 2.5 mg/L. The amount of cholesterol adsorbed in the IMS was found as 83.07 mg/g polymer, which indicates that 92% of the cholesterol in the medium was adsorbed. The selectivity behavior of the cholesterol imprinted polymer was carried out with the stigmasterol and estradiol molecules, which are similar in structure, molecular weight, and character to the cholesterol molecule. The chol-imprinted polymeric beads were 21.38 and 10.08 fold more selective for cholesterol compared to estradiol and stigmasterol steroids used as competitor agents respectively. Kinetic and isotherm calculations of the synthesized cholesterol imprinted polymer were made and reusability experiments were carried out.

## 1. Introduction

The data display that cardiovascular diseases are one of the diseases that cause the most deaths. Scientific studies have shown that cardiovascular diseases are associated with high cholesterol levels [1,2]. For this purpose, studies to reduce the total cholesterol level are still ongoing. The most commonly used drugs to reduce total cholesterol levels are statins. Statins reduce total cholesterol levels by reducing cholesterol production in the liver. However, it is known that they have serious side effects, especially on the liver. Another promising group of drugs is cholesterol adsorptive resins aiming to lower total cholesterol levels. The focal point of these drugs is to prevent the absorption of cholesterol which is taken with the diet and constitutes 25% of total cholesterol. These drugs, which are prescribed with the trade names of Ezetibime and Cholestiramine, prevent the absorption of these cholesterol and bile acids from the small intestines and ensure their excretion with the feces [3,4]. For example, Colesevelam is a commercially prescribed nonabsorbable polymeric resin that reduces intestinal cholesterol absorption [5–7]. In this study, the polymer synthesized from a hydrophobic amino acid can be evaluated for a similar purpose. Polymeric microbeads were synthesized using the MIP technique in order to gain high affinity and selectivity against cholesterol. To demonstrate the operability of the synthesized polymeric microbeads as a cholesterol-binding resin, experimental studies were carried out in intestinal mimicking solution.

Cholesterol plays a role in different functions in the body such as the synthesis of bile acids to digest fats, cell membrane functions, hormones, and vitamin D production. Cholesterol is a necessary substance for a healthy life, as it is found in all cells of our body and forms the basic building block of hormones. However, if cholesterol is above a certain level, it threatens health. Cholesterol combines with bile pigments and causes the formation of gallstones [8,9]. Studies demonstrated that serum cholesterol levels are correlated with cardiovascular diseases which are one of the main reasons for morbidity and mortality in developed countries. If cholesterol is found in the blood in higher than normal amounts, it accumulates in the vein walls and causes narrowing and hardening of the arteries (atherosclerosis). This situation leads to coronary heart diseases, such as peripheral vascular disease, atherosclerosis, stroke, and death [10–13].

Molecular imprinting technique is the method of creating sensitive recognition sites in synthetic polymers by mimicking the recognition methods of biological systems. In the molecular imprinting technique, while specific recognition sites are created, weak intermolecular interactions such as hydrogen bonding and electrostatic hydrophobic interactions are used [14]. In this technique, polymerization is carried out when the analyte (target), functional monomers, and cross-linkers are all together. After the polymerization process is completed, analyte-specific cavities are created in the polymeric matrix by removing the analyte with suitable solvents. In addition to size and shape memory for the target molecule, these distinctive cavities also have chemical functional groups complementary to the template. Polymeric materials prepared using the molecular imprinting method are suitable to apply in many fields such as purification and separation technologies, catalysis, chromatography in sensors, and drug release [15–18].

Many methods are used in the separation and purification of molecules from complex environments. Solid phase extraction is the most widely preferred of these methods. In solid phase extraction, separation is based on the adsorption of analyte molecules onto a solid matrix. In this method, polymeric matrices are more preferred as they allow surface functionality. A highly selective affinity sorbent is prepared by covalent attachment of the ligand with a spacer arm to the polymeric matrix [19–20]. The ligand is selected by considering the chemical properties of the analyte (size, charge, and hydrophobicity, etc.). Pseudo-specific affinity adsorbents are systems in which the ligand is in the structure of the polymeric matrix. Pseudo-specific affinity adsorbents are more advantageous because they do not have spacer arm attachment to the adsorbent and there is no ligand binding step. This approach has many advantages compared with conventional techniques that necessitate the activation of the matrix for ligand immobilization [21]. Here, MATyr behaves as a hydrophobic ligand, and there is no demand for the activation step for ligand immobilization to the matrix. The ligand leakage problem was also overcome with this method.

In this study, biocompatible, hydrophobic poly(HEMA–MATyr) microbeads with high selectivity were synthesized as an alternative to polymeric resins in order to remove the cholesterol molecule from the gastrointestinal tract by using a molecular imprinting technique. In order to selectively remove of hydrophobic cholesterol molecule, the hydrophobic MATyr amino monomer was selected as the functional monomer due to its similarity to the cholesterol molecule and an amino acid component of the cholesterol receptor [22]. Cholesterol imprinted polymers were prepared in approximately 30 μm size by suspension polymerization method. In order to determine the cholesterol adsorption behavior of the synthesized polymer in the natural environment, adsorption experiments were performed in intestinal intestinal-mimicking solution.

## 2. Materials and methods

### 2.1. Materials

Cholesterol (Chol), stigmasterol, estradiol, L-tyrosine methylester, methacryloyl chloride, Hydroxyethyl methacrylate (HEMA), and ethylene glycol dimethacrylate (EGDMA) were purchased from Sigma (St. Louis, USA). Methanol and acetonitrile used in high-performance liquid chromatography (HPLC) analysis were HPLC grade and obtained from Merck A.G. (Darmstadt, Germany). The other chemicals were with the highest purity commercially available. All water used in the experiments was ultrapure and obtained from Barnstead (Dubuque, IA, USA) RO pure LP system.

### 2.2. Preparation of N-methacryloyl-L- tyrosine methylester (MATyr)

**Figure 1:**
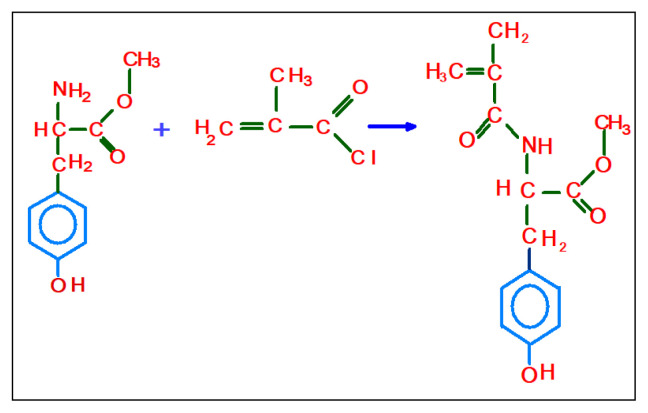
Schematic presentation of synthesis of MATyr monomer from L-tyrosine aminoacid methylester.

In order to imprint cholesterol, N-methacryloyl-L-tyrosine methylester (MATyr) was chosen as a functional monomer due to the similar chemical structure. The synthesis of MATyr as in literature [23] can be given briefly as follows: After dissolving 5 g of L-tyrosine methylester and 0.2 g of hydroquinone in 100 mL of dichloromethane, the obtained mixture was cooled down to 0 °C, and 12.7 g triethylamine was poured to this mixture. After adding of five mL of methacryloyl chloride into the mixture, it was mixed continuously for 2 h magnetically. Lastly, unreacted chemicals were removed by using a 10% NaOH solution. The aqueous phase was evaporated, after that MATyr monomers were firstly crystallized in an ether-cyclohexane mixture and then dissolved in ethyl alcohol. Figure 1 shows a chemical synthesis of the MATyr monomer.

### 2.3. Preparation of precomplex

For preparation MATyr-cholesterol complex, cholesterol was dissolved in methanol, and after that MATyr was put into this solution. Here, MATyr and cholesterol were complexed at the 2:1 molar ratio at 25 °C temperature for 45 min (100 rpm) in methanol.

It is believed that weak intermolecular interactions such as π–π interaction, hydrophobic interactions, and hydrogen bond interactions play a role in the formation of complexes between cholesterol and MATyr monomer due to the chemical structures and functional groups of the molecules. The recognition cavities on the surface of polymeric beads were obtained through these interactions.

### 2.4. Preparation of cholesterol-imprinted microbeads

**Figure 2:**
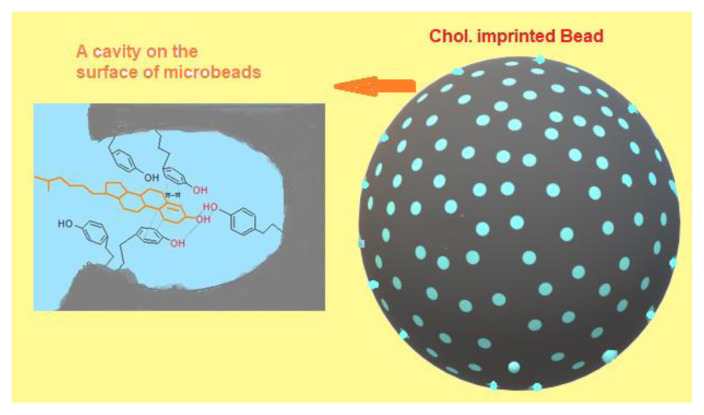
Schematic illustration of cholesterol-adsorbent interaction at created cavity on the surface.

The polymerization recipe may be summarized as below. The poly(vinyl alcohol)(PVAL) as stabilizer was dissolved in 50 mL of deionized water in order to prepare the aqueous phase. By mixing HEMA (4.0 mL), EGDMA (8.0 mL), and toluene as a pore former (12.0 mL) in a test tube, the organic phase was prepared. After preparing cholesterol-MATyr precomplex, the pre-complex was also transferred to the organic monomer phase into the test tube. Benzoyl peroxide (BPO) (100 mg), the initiator, was dissolved in this homogeneous solution. The organic phase that contains precomplex was poured into the aqueous phase in the polymerization reactor (100 mL) which was placed in the water bath equipped with a temperature-control system. After the system temperature was brought to 65 °C, the polymerization solution was stirred at 600 rpm for 30 min. The polymerization was performed at 65 °C for 4 h and at 90 °C for 2 h. After the polymerization was completed, the system temperature was brought to room temperature. The microbeads obtained after the polymerization process were washed with methanol/water solution (70/30, v/v) for 24 h to remove unreacted monomers and chemicals in the medium. Similar polymerization processes were applied in the synthesis of cholesterol nonimprinted polymer by only adding MATyr monomer instead of cholesterol-MATyr pre-complex. Washed microbeads were dried at room temperature in a desiccator. Figure 2 shows the schematic illustration of cholesterol adsorbent interaction at the created cavity on the surface.

For removing the template from microbeads, a warm chloroform solution is used. Cholesterol- imprinted microbeads are added into 15 mL chloroform and mixed for 48 h at 50 °C. The washing process was continued until the leakage of cholesterol molecules from the particles into the medium ceased. After the removal of the template from microbeads, it was cleaned with ethanol/water in a magnetic stirrer at room temperature for 12 h.

### 2.5. Characterization studies of microbeads

Cholesterol-imprinted microbeads were characterized by FTIR-ATR. The infrared (IR) spectra were collected by an ATR Fourier transform IR (FTIR) spectrometer (Bruker, Tensor II, Bruker Optics GmbH., Germany) to identify the functional groups followed by the relevant wavenumber interval of 4000–450 cm^−1^.

The shapes and the surface morphology of synthesized microbeads were analyzed using scanning electron microscopy (SEM) (Carl Zeiss Microscopy GmbH 73447 Oberkochen Germany). For this purpose, the synthesized beads were dried at room temperature for five days before being analyzed. After that a small amount of the sample was covered by sputtering gold under a vacuum and then their images were taken by scanning at desired magnifications.

### 2.6. Adsorption studies

Cholesterol adsorption experiments were carried out in a batch experimental setup. The adsorption studies of cholesterol on the chol-imprinted and nonimprinted beads were carried out with 30 mg polymeric beads in methanol at different cholesterol solution concentrations (0.5–3.5 mg/mL) for 2h.

The solution was centrifuged after adsorption to precipitate the microbeads. The amount of cholesterol in the medium was measured using the HPLC device. HPLC system of an LPG-3000 pump, WPS-3000 autosampler, TCC-3000 column department, PDA-3000 detector and column (Chromasil 100–5, Length/I.D; 150/4.6 mm). Using ABC, a linear gradient was begun and progressed with increasing B from 10% to 65% and decreasing C from 80% to 35% in 1 min and completed in 20 min 100 mL of cholesterol solution was inserted into the column. The absorbance was measured at 280 nm for cholesterol.

The amount of cholesterol adsorbed per unit mass of the polymer beads was calculated by using the following expression:


(1) $$Q=[(C_{0}-C).V)]/\text{m}$$

Here, Q is the amount of cholesterol adsorbed onto the unit mass of the polymer (mg/g); C_o_ and C are the amount of initial and final cholesterol in solution after treatment respectively (mg/mL); V is the volume of the cholesterol solution (mL); and m is the mass of the polymeric beads used (g).

### 2.7. Adsorption studies in intestinal mimicking medium

Intestinal Mimicking solutions (IMS) were prepared to investigate the adsorption behaviors of chol-imprinted microbeads in an environment that closely resembles the natural medium. Firstly, the IMS solution was prepared by mixing 125 mL of 0.2 M potassium dihydrogen phosphate (KH_2_PO_4_) solution and 95 mL of 0.2 M sodium hydroxide (NaOH) solution. The end volume of this mixture solution was brought up to 450 mL by the addition of purified water. Then 24.5 g of sodium deoxycholate (NaDC) and 16.5 g of sodium cholate (NaC) were added to this solution. The pH of the solution was adjusted to 7.4 and the volume of the solution was completed to 500 mL and the solution was kept in a dark room. Nine hundred mg of cholesterol was added to the prepared IMS solution and the solution was sonicated for 4 h at 50 ºC and made ready for use [24].

Adsorption studies were carried out by adding 30 mg of dry chol-imprinted microbeads into 5 mL of cholesterol standard solution. Then, the samples were mixed with a magnetic stirrer at room temperature for 24 h. The solution was centrifuged to precipitate the beads, and the cholesterol amount in the supernatant was measured by HPLC which was described above. All the experiments and the analysis were repeated three times.

### 2.8. Reusability and selectivity studies

The selectivity of the synthesized beads for cholesterol was investigated against estradiol and stigmasterol due to their very similar structure, molecular weight, and hydrophobic character. Selectivity studies were carried out by adding 30 mg of polymeric beads to the 5 mL of water/tetrahydrofuran (THF) mixture (5:6 v/v) of estradiol and stigmasterol. The mixture solution was stirred on a magnetic stirrer at room temperature for 2 h. After centrifugation, microbeads were removed from the medium. Analysis of steroids adsorbed to microbeads was performed by HPLC. The adsorbed steroid amount was computed from the difference between the initial and the final result.

The distribution coefficients (K_d_) for stigmasterol and estradiol with respect to cholesterol were calculated by using Eq. (2):


(2) $$K_{d}=[(C_{i}-C_{f})/C_{f}].\text{V}/\text{m}$$

Herein, K_d_ represents the distribution coefficient for any streoids (mL/g); C_i_ and C_f_ are the initial and final concentrations of analyts (mg/mL), respectively. V is the volume of the IMS solution (mL) and m is the weight of dry microbeads (g).

The imprinting factor (k) for the binding of cholesterol in the presence of the competing agents stigmasterol and estradiol was calculated by Eq. (3),


(3) $$k=K_{d(cholesterol)}/K_{d(competitor)}$$

A comparison of the *k* values of chol-imprinted microbeads allows an estimation of the effect of imprinting on selectivity. The imprinting factor (*k′*) is an indicator of the competitive binding efficiency of imprinted bead recognition sites in comparison with nonimprinted. It can be defined as;


(4) $$k^{\prime }=k_{imprinted\;polymers}/k_{non-imprinted\;polymers}$$

Desorption of cholesterol molecules from microbeads was carried out with chloroform/ethanol solution. For the desorption process, the chol-imprinted beads were put into the desorption solution and mixed for 12 h at 400 rpm. The final cholesterol concentration in the desorption medium was analyzed by HPLC. In order to define the reusability of cholesterol-imprinted microbeads, the adsorption-desorption process was performed at least 10 times using the same microbeads. Chol-imprinted microbeads were washed with 50 mM NaOH solution after the regeneration and sterilization process of each cycle.

## 3. Results and discussion

### 3.1. Characterization of cholesterol imprinted microbeads

**Figure 3:**
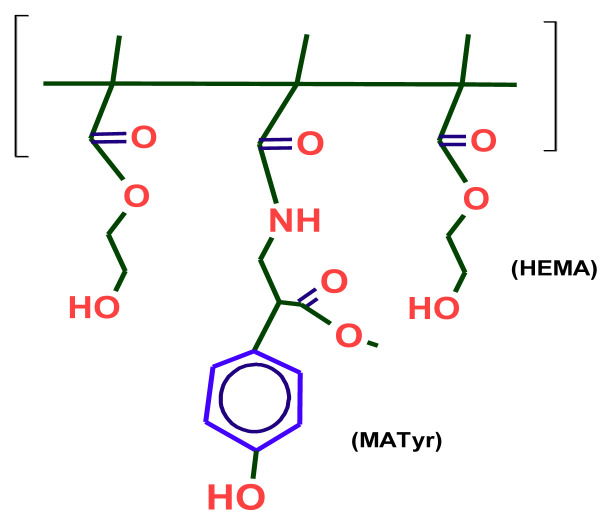
The chemical structure of the poly(HEMA–MATyr) microbeads.

The chemical structure of chol-imprinted poly(HEMA-MATyr) microbeads is shown in Figure 3.

**Figure 4:**
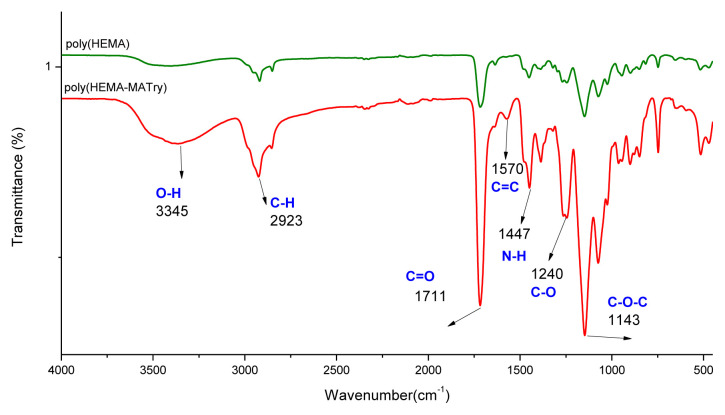
FTIR spectra of poly(HEMA-MATyr) microbeads.

According to the spectrum of poly(HEMA-MATyr) microbeads in Figure 4, the peaks around 3345 cm^−1^ are for -OH while the peaks at 1711 cm^−1^ and 2923 cm^−1^ correspond C=O and C-H stretching, respectively. The characteristic stretching vibration amide I and amide II absorption bands were at 1570cm^−1^ and 1447cm^−1^ as shown in Figure 4. Furthermore, the spectrum of poly(HEMA-MATyr) microbeads indicates two peaks at 1143 and 1240 cm^−1^ corresponding to the stretching vibrations of the ether (C–O–C) and carboxylate (CO-O) groups, respectively.

**Figure 5:**
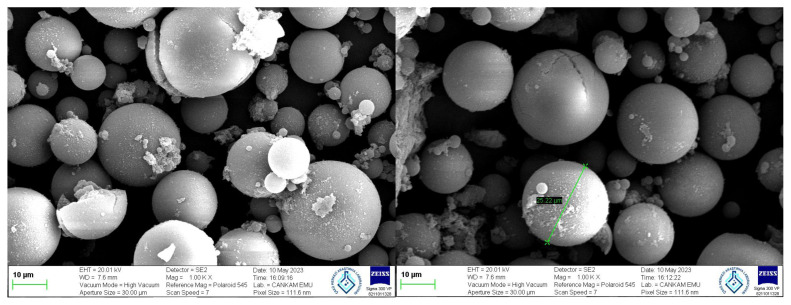
SEM micrograph of poly(HEMA-MATyr) microbeads.

From the scanning electron micrograph (SEM) photographs of the chol-imprinted poly(HEMA-MATyr) microbeads given in Figure 5, it can be seen that the microbeads are spherical and 30 μm in diameter. The chol-imprinted poly(HEMA-MATyr) microbeads are hydrophobic character and have highly cross-linked polymer networks. These highly cross-linked properties provide to preserve the three-dimensional shape of the recognition sites on the surface and remain insoluble spherical microbeads.

### 3.2. Adsorption studies

The cholesterol adsorption studies were carried out with 5 mL cholesterol solution in batch system in methanol at room temperature. Thirty mg dried chol-imprinted polymeric beads were added to the adsorption medium. After adsorption, the solution was centrifuged at 5000 rpm for 10 min to precipitate microbeads. The cholesterol was determined in the supernatant after the removal of the polymeric beads. At the end of this adsorption process, the amount of cholesterol adsorbed to the polymer was determined by taking the difference between the initial and final values of the amount of cholesterol measured using HPLC as mentioned before.

**Figure 6:**
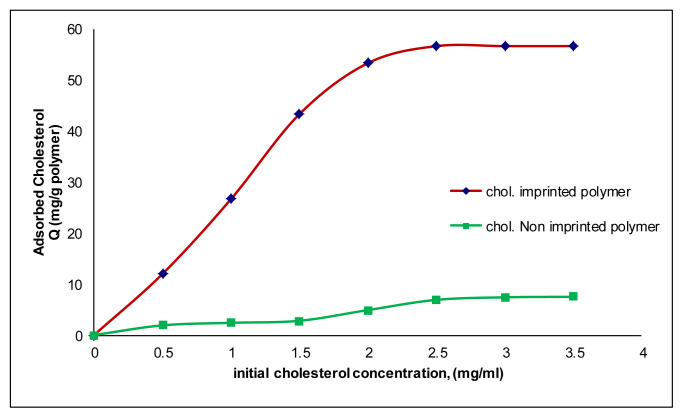
Effect of cholesterol initial concentration on cholesterol adsorption onto chol-imprinted poly(HEMA-MATyr) microbeads.

The cholesterol adsorption capacity of the synthesized polymer was determined by using cholesterol solutions at different concentrations (0.5–3.5 mg/mL). As can be seen from Figure 6, the amount of cholesterol adsorbed to the polymer increases depending on the increase in the amount of cholesterol in the adsorption medium. The maximum adsorption capacity of microbeads was determined as 56.67 mg/g at a concentration of 2.5 mg/mL. The cholesterol binding capacity of chol-imprinted microbeads was higher than that of nonimprinted microbeads because of their selective recognition cavities. The negligible amount of cholesterol molecules (7.5 mg/g polymer) adsorbed on the nonimprinted microbeads.

**Figure 7:**
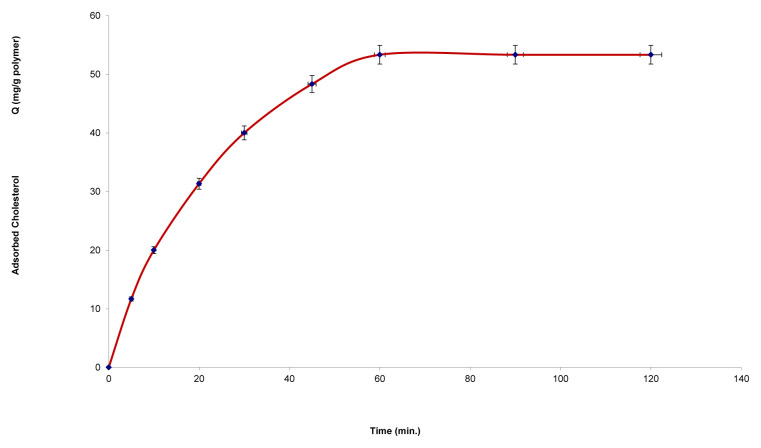
Effect of cholesterol contact time on cholesterol adsorption onto chol-imprinted poly(HEMA-MATyr) microbeads.

Figure 7 shows the effect of contact time on the adsorption of the cholesterol onto the microbeads. The binding amount of cholesterol was increased depending on the increase in the contact time of cholesterol with the hydrophobic adsorbent. As can be seen in Figure 7, the maximum adsorption capacity was reached at approximately 60 min.

### 3.3. The adsorption of cholesterol from gastrointestinal mimicking solution

Studies to determine the ability of the synthesized chol-imprinted microbeads to bind cholesterol molecules in the small intestine, as well as their chromatographic or efferent therapy uses, were performed in IMS solution. To create an environment close to the intestinal environment, bile acids were added to the medium and the pH of the solution was made basic by adding NaOH solution. Experimental studies were carried out in a batch experimental system and the solution temperature was kept constant at 37 °C in order to be close to in vivo studies. After adding microbeads to the solution, cholesterol adsorption was allowed by stirring in a magnetic stirrer for 4 h.

At the end of the adsorption process, the microbeads were removed by centrifugation and the amount of adsorbed cholesterol by the microbeads was calculated by subtracting the amount of cholesterol in the final solution from the amount of cholesterol in the initial solution. The amount of cholesterol adsorbed in the IMS was found to be 83.07 mg/g polymer, which indicates that 92% of the cholesterol in the medium was adsorbed. The high amount of adsorbed cholesterol may be due to the fact that the natural environment provides more opportunities for the molecule to reach the cavities where cholesterol interacts.

### 3.4. Selectivity studies

**Figure 8:**
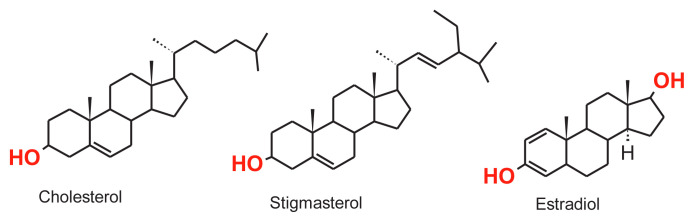
Chemical structures of competitive agents with cholesterol stigmasterol and estradiol.

Selectivity studies of hydrophobic microbeads prepared using a molecular imprinting technique to have high selectivity against cholesterol were performed in the presence of estradiol and stigmasterol molecules. These molecules, whose chemical formulas are given in Figure 8, were chosen as competitive agent because their molecular weights, structural and hydrophobic chemical characters are similar to cholesterol.

In the study, while the polymer was designed, it was aimed to create specific cavities for cholesterol molecules on the surface of the polymeric matrix by molecular imprinting method. It was planned that the synthesized polymer could selectively adsorb cholesterol molecules using hydrophobic, hydrogen bonding, and π–π weak interactions.

**Figure 9:**
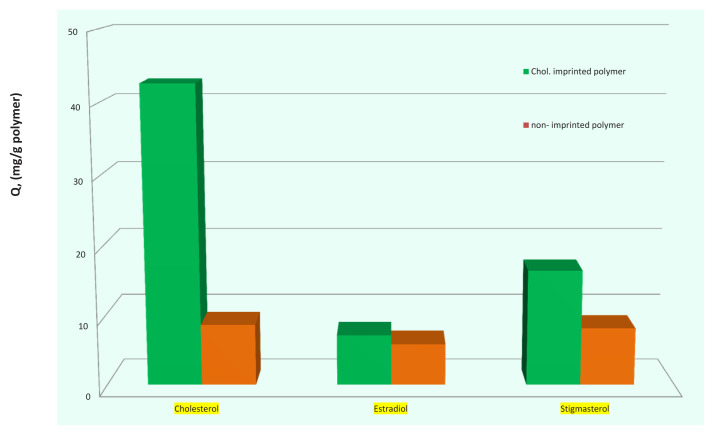
Cholesterol adsorption of chol-imprinted and nonimprinted poly(HEMA-MATyr) microbeads in the presence of estradiol and stigmasterol molecules.

In Figure 9, the adsorption behaviors of chol-imprinted and nonimprinted microbeads in the presence of competitive agents added to the same adsorption medium together with the cholesterol molecule are given. As it is indicated in the figure, the amount of adsorption of cholesterol molecules by the polymeric chol-imprinted microbeads from the mixed medium is quite higher than the other molecules in the medium. This shows that the imprinting technique has been successfully applied and cholesterol-specific cavities have been created on the polymeric matrices. It is seen that the amount of estradiol adsorbed is lower due to the hydrophilic character than stigmasterol. In addition, although the adsorbing of the analyte molecules to the NIP polymers has occurred, it is seen that it is in very low amounts.

**Figure 10:**
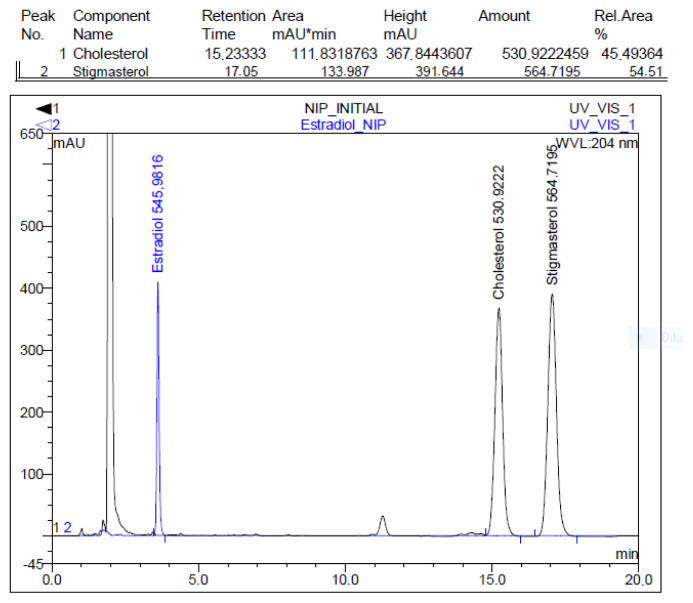
The HPLC chromatogram of cholesterol and competitor agents.

Figure 10 shows an HPLC chromatogram of the selectivity experiments performed with the mixture of cholesterol stigmasterol and estradiol molecules to determine the selectivity behavior of the synthesized polymeric microbeads.

Table 1 shows the selectivity coefficients of chol-imprinted polymers against cholesterol and competitor molecules. According to Table 1, the Kd value of cholesterol molecule for the chol-imprinted beads was higher than the Kd value of the competitor steroids. And, the Kd values of chol-imprinted polymers were also higher than nonimprinted polymers. The results show that chol-imprinted beads have more cholesterol adsorption capacities than either stigmasterol or estradiol. As can be seen from the table, the cholesterol selectivity of imprinted polymeric beads in comparison with nonimprinted beads are 21.38 and 10.08 times more selective than estradiol and stigmasterol respectively.

### 3.5. Adsorption kinetic study

Adsorption kinetics is a crucial issue in understanding the adsorption steps that affect the rate of the adsorption process. The adsorption process takes place with a multistep mechanism, including diffusion of the analyte to the adsorbent surface by mass transfer, diffusion into the micro pore, and physical or chemical binding of the analyte to the adsorbents. Pseudo-first-order means diffusion-controlled and pseudo-second-order also means chemically-controlled kinetic models express this multistep mechanism. While the pseudo-first-order kinetic model indicates that the step determining the adsorption rate is the diffusion of the analyte to the adsorbent surface, the pseudo-second-order kinetic model explains that the rate-determining step is the interaction between the analyte and adsorbent.

First and second-order kinetic models were implemented to experimental data to determine the adsorption rate step. Thus, it was determined that the adsorption rate step is mass transfer or chemical reactions. The pseudo-first-order velocity equation, also known as the Lagergren first-order velocity equation, is expressed by the following equation:


(5) $$\left(\Delta \;Q_{t}/d_{t})=k_{1}(Q_{e}-Q_{t}\right)$$

In the equation, k_1_ represents the pseudo-first-order adsorption rate constant (min^−1^), Q_e_ and Q_t_ represent the amount of molecules adsorbed at equilibrium time and at any time t (mg/g), respectively.

Applying and integrating the boundary conditions Q_t_ = 0 at t = 0 and Q_t_ = Q_t_ at time t = t;


(6) $$log[Q_{e}/(Q_{e}-Q_{t})]=(k_{1}t)/2.303$$

gives the equality. Equation 6 linearized by rearrangement:


(7) $$log(Q_{e}-Q_{t})=\text{log}(Q_{e})-(k_{1}t)/2.303$$

The linearity of the log(Q_e_) versus t plot shows the applicability of the kinetic model. In true first-order operation, log(Q_e_) should equal log(Q_e_-Q_t_) versus the cutoff point of the t-graph. In addition, the pseudo-second-order equation based on the adsorption equilibrium capacity can be given as:


(8) $$\Delta \;Q_{t}/d_{t}=k_{2}{\left(Q_{e}-Q_{t}\right)}^{2}$$

In the equation, k_2_ is the pseudo-second-order rate constant (g.mg^−1^.min^−1^). By applying the boundary conditions Q_t_ = 0 at t = 0 and qt = qt at t = t to Equation 8;


(9) $$Q_{t})=(1-Q_{e})+k_{2}t$$

equality is achieved. The linear version of this equation is:


(10) $$(t/\;Q_{t})=(1/k_{2}{Q_{e}}^{2})+(1+Q_{e})t$$

is expressed with Equation. 10. In order to second-order kinetics to be applicable, the t/Q_t_ versus t plot must be linear. The rate constant (k_2_) and equilibrium adsorption (Q_e_) can be obtained from the cutoff point and slope, respectively.

The pseudo-first and second-order kinetic constants of chol-imprinted microbeads are given in Table 2. As a result of the calculations, it is seen that the second-order kinetic model is more suitable for cholesterol adsorption in methanol medium by chol-imprinted microbeads. The theoretical Q_e_ values obtained in the second-order kinetic calculations are quite close to the experimental Q_e_ values. These results show that the adsorption was performed chemically controlled. That is, the adsorption behavior conforming to the pseudo-second-order kinetic model shows that the diffusion restrictions are negligible. Thus, the chemical adsorption that is the specific binding reaction between cholesterol and the MATyr hydrophobic functional group controls the kinetic behavior.

### 3.6. Adsorption isotherm study

Adsorption isotherms represent the equilibrium state between the analyte in solution and the analyte adsorbed on the surface. Adsorption equilibrium is the situation in which the amount of analyte adsorbed on the adsorbent is equal to the amount of analyte desorbed from the surface. Adsorption isotherms provide information about the adsorption mechanism, surface properties, and affinity of the adsorbent. In order to determine the adsorption mechanism and explain the experimental data, widely applied Langmuir and Freundlich adsorption models were used in this study. The Langmuir adsorption isotherm presumes homogeneous, single-layer adsorption, as well as uniform or equivalent adsorption with no lateral interactions and no steric hindrance between adsorbed molecules. The Freundlich isotherm defines nonideal and reversible adsorption that is not limited to monolayer adsorption. The Langmuir isotherm model assuming homogeneous binding is widely used because it fits the interactions of molecularly imprinted adsorbents.

The Freundlich adsorption model is particularly suitable for molecular imprinting systems at low concentrations. However, this model shows some deviations at high concentrations. Equations (11) and (12), represent the functions of Langmuir and Freundlich isotherms, respectively.


(11) $$Q_{e}=Q_{max}.b.C_{e}/(1+b.C_{e})$$

At this Langmuir isotherm equation, Q_e_ is the Langmuir monolayer rebinding capacity (mg/g), C_e_ is the equilibrium cholesterol concentration (mg/mL), and b is the constant concerned with the affinity binding sites. A linearized plot of C_e_/Q_e_ versus C_e_ enables to calculation of the values of Q_max_ and b, in turn.


(12) $$Q_{e}=K_{F}.{C_{e}}^{1/n}$$

**Figure 11:**
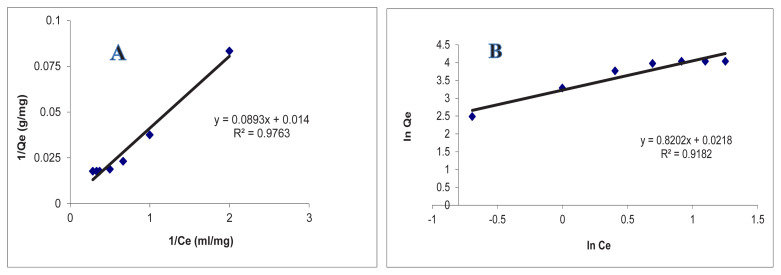
Langmuir (A); Freundlich (B) adsorption isotherm models.

K_F_ and 1/n can be identified from the linear plot of lnQ_e_ versus ln C_e_. Herein, K_F_ is the Freundlich constant and it roughly indicates the rebinding capacity. The Freundlich exponent (1/n) represents the heterogeneity of the system. More homogeneous systems will have an n value approaching unity whereas more heterogeneous systems will have an n value approaching zero. Graphics of Langmuir and Freundlich models are given in Figure 11.

When the correlation coefficient (R) values in Table 3 are examined, it has been observed that the interaction between the chol-imprinted polymeric adsorbent and cholesterol molecules best fits the Langmuir isotherm model. The Langmuir isotherm model had the highest R^2^ value compared to the Freundlich isotherm models. This result shows the binding properties of cholesterol molecules to the prepared chol-imprinted polymeric adsorbent surface are monolayer, have equal energy, minimum lateral interaction, and are homogeneously distributed.

### 3.7. Desorption and reusability studies

Although these polymers are synthesized with the aim of adsorbing cholesterol molecules from the digestive tract, desorption, and regeneration studies were also carried out, since they can also be used in chromatographic studies.

**Figure 12:**
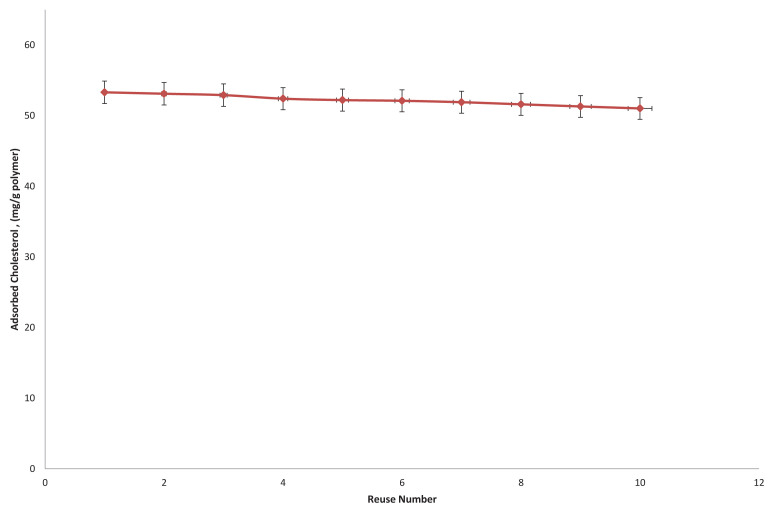
Reusability of chol-imprinted poly(HEMA-MATry) microbeads.

The regeneration and reusability studies of the synthesized microbeads were performed in the batch system for 2 h and at room temperature. For this purpose, adsorption and desorption studies were applied to the same beads 10 times. Cholesterol adsorbed to the chol-imprinted polymers were desorbed using 50 mL of chloroform/Ethanol (1/2,v/v) desorption solution. In desorption studies, it is seen that cholesterol adsorbed by chol-imprinted polymers can be desorbed by 96%. It can be seen from Figure 12 that there is no significant reduction in the cholesterol adsorptive capacity of the polymers at the end of the adsorption-desorption process repeated 10 times.

### 3.8. Comparison with related literature

Because of the negative effects of cholesterol on human health, there are many studies on cholesterol in the literature. These studies are aimed at removing cholesterol from different environments with adsorbents prepared with different methods which have different sizes and shapes. As well as studies of reducing cholesterol from human blood [25–28] and removal from the intestinal system [22,29,30], these studies include studies of removing cholesterol from different animal-based foods such as milk [30–33] and eggs [32]. Due to the hydrophobic character of cholesterol, adsorbents generally contain ligands based on hydrophobic interaction. Studies in the related literature have focused on increasing the adsorption capacity and selectivity of adsorbents, and the adsorbents are mostly prepared by using molecular imprinting technique [34–43] and immunoaffinity ligand bonded adsorbents. Adsorbents prepared from polymers or composites are in the form of membranes [29,38], micro/nano beads [24,26,28,30,33,35,43], or cryogels [27,31,44]. Experimental studies to reduce cholesterol levels have been carried out in different adsorption media such as human serum plasma, methanol, hexane, IMS, and milk. New and different studies in the literature and data on adsorption capacities are summarized in Table 4.

In this experimental study, microbeads were synthesized by molecular imprinting method using MATyr monomer, a hydrophobic pseudo-specific affinity ligand for adsorption based on hydrophobic interaction, and the cholesterol molecule was successfully removed from the IMS solution with high selectivity and capacity.

## 4. Conclusion

The selective separation of biological molecules from their natural environments has been a subject that scientists have been focusing on for a long time. Adsorbents have been synthesized by suitable methods in order to separate with high selectivity the target molecules from complex environments. While developing adsorbents specific to the target molecule in separation/purification or removal processes, the chemical properties of the molecule are analyzed in detail. In these processes, molecule-specific properties such as size, charge, or hydrophobicity are taken into consideration. The molecular imprinting method is a method used in the preparation of adsorbents that selectively recognize the target molecule in the separation/purification, removal, or detection processes of biological molecules. Today, adsorption-based polymeric resins are used to keep the cholesterol molecule at a certain level in order to protect human health.

In this study, biocompatible, hydrophobic microbeads with high selectivity were synthesized as an alternative to polymeric resins in order to remove the cholesterol molecule from the gastrointestinal tract by using molecular imprinting technique. It is thought that intermolecular secondary interactions are used based on the adsorption of the cholesterol molecule to the polymer. There are hydrophobic interactions, π–π interactions, and hydrogen bond-based interactions between the adsorbent, which is a biocompatible polymer based on HEMA and containing tyrosine amino acid, and the cholesterol molecule. Adsorption studies of synthesized polymeric adsorbent on cholesterol molecules were carried out in methanol and intestinal mimicking solution. The selectivity of chol-imprinted microbeads to the cholesterol molecule was determined using stigmastrole and estradiol molecules, which are very similar in structural and chemical characteristics to the cholesterol molecule. In the selectivity studies, it was observed that the synthesized micros beads showed 21.38 times higher affinity for cholesterol molecules than estradiol and 10.08 times higher than stigmasterol.

This study will provide a new perspective to the scientists working in this field, as well as making serious contributions to the literature, with the adsorbent, which has a high affinity and selectivity to the cholesterol molecule and may be an alternative, compared to the polymeric resins developed to prevent the entry of cholesterol into the body by adsorption from the intestinal environment. In addition to making serious contributions to the literature, with the adsorbent, which has a high affinity and selectivity to the cholesterol molecule and may be an alternative to the polymeric resins that prevent cholesterol from entering the body through adsorption from the intestinal environment, this study will provide a new perspective to the scientists working in this field.

## Figures and Tables

**Table 1:** Selectivity coefficients and relative selectivity.

Polymer					
Competitive molecules	Chol. nonimprinted polymer		Chol-imprinted polymer		
	k_d_	K	k_d_	K	k′
**Cholesterol**	25.75		424.24		
**Estradiol**	56.54	0.45	43.55	9.74	21.38
**Stigmasterol**	72.84	0.35	118.93	3.56	10.08

**Table 2:** The first-order and second-order kinetic constants for chol-imprinted microbeads.

Initial Conc.	Experimental	First-order kinetic			Second-order kinetic		
(mg/mL)	Q_e_ (mg/g)	k_1_ (1/min)	Q_e_ (mg/g)	R^2^	k_2_ (1/min)	Q_e_ (mg/g)	R^2^
2.5	56.67	0.072	46.08	0.9159	0.00174	48.08	0.9811

**Table 3:** The Langmuir and Freundlich constants for chol-imprinted microbeads.

Exp.	Langmuir constants			Freundlich constants		
Q_ex_ (mg/g)	q_max_ (mg/g)	b (mL/mg)	R^2^	K_F_ (mg/g)	n	R^2^
56.67	71.42	0.15	0.97	18.83	1.22	0.92

**Table 4:** Comparison of the cholesterol adsorption efficacy of different adsorbents.

Material/Adsorbent	Medium	Max. adorp. Q(mg/g) or Removal (%)	Reference
P(HEMA-MATyr)	Artificial human plasma	714.2/ 95.3%	[25]
Chol. imprinted granular polymers by co-polymerization on the surface of selenium nanoparticles	Blood plasma in vitro	40.2%	[26]
Random and oriented anti-LDL antibody immobilized p(HEMA) cryogel	Hypercholesterolemic plasma	111 and 129 mg	[27]
Immunoaffinity poly(HEMA-EGDMA) beads	hypercholesterolemic human plasma	28.3	[28]
PHEMA-Chol./MIP	IMS	23.2	[22]
Chol. imprinted poly(HEMA-methacryloyloamidotryphan) particles embedded composite membrane	IMS	9.24	[29]
P(HEMA-MAPA)-Chol.-MIP Nanospheres	IMS	714.17	[30]
PGMA-Chol-MIP embedded PHEMA Cryogel	Milk and IMS	42.7 %80	[31]
Fe_3_O_4_ nanoparticles/-β-Cyclodextrin	Milk and egg yolk	208.3	[32]
β-cyclodextrin	Milk	94.3%	[33]
Cholesteryl chitin carbonate (Chol-Chi). Sacrifice spacer	Methanol	13.6	[34]
Molecularly imprinted polymercore–shell superparamagnetic Fe3O4 nanoparticle	Toluen	20.1	[35]
Silica-Cyclodextrin-Chol/MIP	Ethanol	76.5	[36]
Poly(methacrylic acid)/silica (PMAA–SiO2) hybrid material Organik-Iorganic polymer	Hexane	113.3	[37]
Methylmethacrylate/acrylic acid Adsorption on nanoparticlescontain membrane	Phosphate buffer	115.4	[38]
Acrylamide/ferric-ion Bulk Polymerization	Dichloromethane	14.48	[39]
Monocholesteryl itaconate/ethylene glycol dimethacrylate Bulk	Aqueous media	32.5	[40]
Cholesteryl (4-vinyl) phenyl carbonate Bulk	Glacial acetic acid	36.7	[41]
Chol-imprinted monoliths Macroporous MAA-BMA-EDMA and HEMA-BMA-EDMA terpolymers monoliths	Methanol and *n*-hexane	0.56	[42]
Fe_3_O_4_/SiO_2_/MIP	CHCl_3_	314.0	[43]
poly(2-hyroxyethyl methacrylate-N-methacryloyl-L-tryptophan methylester) cryogel beads	Milk	288.72	[44]
Thymus vulgaris L. Powder before and after milling	Human serum	47 and 38%	[45]
Aluminium/MOF-β-Cyclodextrin	Butter and sheep tail	33.07% 2.8	[46]
Coprecipitation, kneading, physical mixture complexation methods with β-cyclodextrin	Butter	91.54; 27.85 and 16.81 %	[47]
Chol-imprinted poly(HEMA-MATyr) microbeads	IMS	**83.07**	**This study**

## References

[1] Sanchis Gomar F Perez Quilis C Leischik R Lucia A Epidemiology of coronary heart disease and acute coronary syndrome Annals of Translational Medicine 2016 4 13 1 12 10.21037/atm.2016.06.33 PMC4958723 27500157

[2] Reiner Ž De Backer G Fras Z Kotseva K Tokgözoglu L EUROASPIRE Investigators Lipid lowering drug therapy in patients with coronary heart disease from 24 European countries--Findings from the Euroaspire IV survey Atherosclerosis 2016 246 243 250 10.1016/j.atherosclerosis.2016.01.018 26812002

[3] Taylor FC Huffman M Ebrahim S Statin therapy for primary prevention of cardiovascular disease Journal of the American Medical Association (JAMA) 2013 310 22 2451 2452 10.1001/jama.2013.281348 24276813

[4] Phan BA Dayspring TD Toth PP Ezetimibe therapy: mechanism of action and clinical update Vascular Health and Risk Management 2012 8 415 427 10.2147/VHRM.S33664 22910633 PMC3402055

[5] Brown L Rosner B Willett WW Sacks FM Cholesterol-lowering effects of dietary fiber: a meta-analysis The American Journal of Clinical Nutrition 1999 69 1 30 42 10.1093/ajcn/69.1.30 9925120

[6] Feingold KR Cholesterol Lowering Drugs Feingold KR Anawalt B Blackman MR Endotext South Dartmouth (MA) MDText.com, Inc 2000

[7] Fonseca VA Rosenstock J Wang AC Truitt KE Jones MR Colesevelam HCl improves glycemic control and reduces LDL cholesterol in patients with inadequately controlled type 2 diabetes on sulfonylurea-based therapy Diabetes Care 2008 31 8 1479 1484 10.2337/dc08-0283 18458145 PMC2494667

[8] Ma H Cholesterol and human health, Nature and Science 2004 2 4 17 22

[9] Tabas I Cholesterol in health and disease The Journal of Clinical Investigation 2002 110 5 583 590 10.1172/JCI16381 12208856 PMC151113

[10] Kratz M Dietary cholesterol, atherosclerosis and coronary heart disease von Eckardstein Arnold Handbook of Experimental Pharmacology Springer Berlin, Heidelberg, Germany Springer 2005 195 213 10.1007/3-540-27661-0_6 16596800

[11] Després JP Lemieux I Dagenais GR Cantin B Lamarche B HDL-cholesterol as a marker of coronary heart disease risk: The Québec cardiovascular study Atherosclerosis 2000 153 2 263 272 10.1016/s0021-9150(00)00603-1 11164415

[12] Fernandez ML Webb D The LDL to HDL cholesterol ratio as a valuable tool to evaluate coronary heart disease risk Journal of the American College of Nutrition 2008 27 1 1 5 10.1080/07315724.2008.10719668 18460475

[13] Alenghat FJ Davis AM Management of Blood Cholesterol Journal of the American Medical Association (JAMA) 2019 321 8 800 801 https://doi:10.1001/jama.2019.0015 30715135 10.1001/jama.2019.0015 PMC6679800

[14] Martín Esteban A Molecularly-imprinted polymers as a versatile, highly selective tool in sample preparation TrAC Trends in Analytical Chemistry 2013 45 169 181 10.1016/j.trac.2012.09.023

[15] Erdem Ö Saylan Y Andaç M Denizli A Molecularly Imprinted Polymers for Removal of Metal Ions: An Alternative Treatment Method Biomimetics 2018 3 4 38 10.3390/biomimetics3040038 31105259 PMC6352701

[16] Say R Erdem M Ersöz A Türk H Denizli A Biomimetic catalysis of an organophosphate by molecularly surface imprinted polymers Applied Catalysis A: General 2005 286 2 221 225 10.1016/j.apcata.2005.03.015

[17] Akgönüllü S Kılıç S Esen C Denizli A Molecularly imprinted polymer-based sensors for protein detection Polymers 2023 15 3 629 10.3390/polym15030629 36771930 PMC9919373

[18] Zabihi S Bakhshpour M Çalışır M Topcu AA Denizli A Preparation of molecular imprinted injectable polymeric micro cryogels for control release of mitomycin C Polymer Bullettin 2023 80 3883 3895 10.1007/s00289-022-04233-y

[19] Lasáková M Jandera P Molecularly imprinted polymers and their application in solid-phase extraction Journal of Separation Science 2009 32 799 812 10.1002/JSSC.200800506 19219838

[20] Shi Y Zhang JH Shi D Jiang M Zhu YX Selective solid-phase extraction of cholesterol using molecularly imprinted polymers and its application in different biological samples Journal of Pharmaceutical and Biomedical Analysis 2006 42 5 549 555 10.1016/j.jpba.2006.05.022 16859856

[21] Garipcan B Denizli A A Novel Affinity Support Material for the Separation of Immunoglobulin G from Human Plasma Macromolecular Bioscience 2002 2 135 144 10.1002/1616-5195(20020401)2:3<135::AID-MABI135>3.0.CO;2-8

[22] Rone MB Fan J Papadopoulos V Cholesterol transport in steroid biosynthesis: role of protein-protein interactions and implications in disease states Biochimica et Biophysica Acta 2009 1791 7 646 658 10.1016/j.bbalip.2009.03.001 19286473 PMC2757135

[23] Yavuz H Karakoç V Türkmen D Say R Denizli A Synthesis of cholesterol imprinted polymeric particles International Journal of Biological Macromolecules 2007 41 1 8 15 10.1016/j.ijbiomac.2006.11.011 17222902

[24] Sellergren B Wieschemeyer J Boos KS Seidel D Chemistry of Materials 1998 10 12 4037 4046 10.1021/cm980730u

[25] Kalburcu T Öztürk N Tüzmen N Akgöl S Denizli A Cholesterol removal onto the different hydrophobic nanospheres: a comparison study Journal of Industrial and Engineering Chemistry 2014 20 153 159 10.1016/j.jiec.2013.04.013

[26] Polyakova I Borovikova L Osipenko A Vlasova E Volchek B Surface molecularly imprinted organic-inorganic polymers having affinity sites for cholesterol Reactive and Functional Polymers 2016 109 88 98 10.1016/j.reactfunctpolym.2016.10.010

[27] Bereli N Şener G Yavuz H Denizli A Oriented immobilized anti-LDL antibody carrying poly(hydroxyethyl methacrylate) cryogel for cholesterol removal from human plasma Materials Science and Engineering: C 2011 31 1078 1083 10.1016/j.msec.2011.03.008

[28] Yavuz H Denizli A Immunoadsorption of cholesterol on protein A oriented beads Macromolecular Bioscience 2005 5 1 39 48 10.1002/mabi.200400068 15635714

[29] Odabaşı M Uzun L Baydemir G Aksoy NH Acet Ö Cholesterol imprinted composite membranes for selective cholesterol recognition from intestinal mimicking solution Colloids and Surfaces B: Biointerfaces 2018 163 266 274 10.1016/j.colsurfb.2017.12.033 29316524

[30] Inanan T Tuzmen N Akgol S Denizli A Selective cholesterol adsorption by molecular imprinted polymeric nanospheres and application to GIMS International Journal of Biological Macromolecules 2016 92 451 460 10.1016/j.ijbiomac.2016.07.007 27411294

[31] Çaktü K Baydemir G Ergün B Yavuz H Cholesterol removal from various samples by cholesterol-imprinted monosize microsphere-embedded cryogels Artificial Cells, Nanomedicine, and Biotechnology 2014 42 365 375 10.3109/21691401.2013.832684 24303869

[32] Sun Y Xu B Mu Y Ma H Qu W Functional magnetic nanoparticles for highly efficient cholesterol removal Journal of Food Science 2018 83 122 128 10.1111/1750-3841.13999 29227533

[33] Lee DK Ahn J Kwak HS Cholesterol removal from homogenized milk with beta-cyclodextrin Journal of Dairy Science 1999 82 11 2327 2330 10.3168/jds.S0022-0302(99)75481-0 10575599

[34] Li X Tong Y Jia L Guan H Fabrication of molecularly cholesterol-imprinted polymer particles based on chitin and their adsorption ability Monatshefte für Chemie 2015 146 423 430 10.1007/s00706-014-1369-4

[35] Zengin A Yildirim E Tamer U Caykara T Molecularly imprinted superparamagnetic iron oxide nanoparticles for rapid enrichment and separation of cholesterol Analyst 2013 138 7238 7245 10.1039/c3an01458d 24133677

[36] Soares CMF Zanin GM Moraes FF Santos OAA Castro HF Molecular imprinting of β-cyclodextrin/cholesterol template into a silica polymer for cholesterol separation Journal of Inclusion Phenomena and Macrocyclic Chemistry 2007 57 79 82 10.1007/s10847-006-9218-7

[37] Wang S Xu J Tong Y Wang L He C Cholesterol-imprinted polymer receptor prepared by a hybrid imprinting method Polymer International 2005 54 1268 1274 10.1002/pi.1841

[38] Ciardelli G Borrelli C Silvestri D Cristallini C Barbani N Supported imprinted nanospheres for the selective recognition of cholesterol Biosensors and Bioelectronics 2006 21 2329 2338 10.1016/j.bios.2005.12.027 16574398

[39] Sreenivasan K Sivakumar R Ferric Iron-Containing Molecularly Imprinted Polymer as an Adsorbent for Cholesterol Adsorption Science Technology 2003 21 3 261 268 10.1260/026361703322404403

[40] Gore M Karmalkar R Kulkarni M Enhanced capacities and selectivities forcholesterol in aqueous media by molecular imprinting: role of novelcross-linkers Journal of Chromatography B 2004 804 211 221 10.1016/j.jchromb.2003.12.028 15093175

[41] Hwang CC Lee WC Chromatographic characteristics of cholesterol-imprinted polymers prepared by covalent and non-covalent imprinting methods Journal of Chromatography A 2002 962 1–2 69 78 10.1016/s0021-9673(02)00559-9 12198973

[42] Stepanova MA Kinziabulatova LR Nikitina AA Korzhikova Vlakh EG Tennikova TB Cholesterol-imprinted macroporous monoliths: Preparation and characterization Electrophoresis 2017 38 22–23 2965 2974 10.1002/elps.201700335 28881397

[43] Effting L Prete MC Urbano A Effting LM González MEC Preparation of magnetic nanoparticle-cholesterol imprinted polymer using semi-covalent imprinting approach for ultra-effective and highly selective cholesterol adsorption Reactive and Functional Polymers Reactive and Functional Polymers 2022 172 105178 10.1016/j.reactfunctpolym.2022.105178

[44] Kartal F Denizli A Molecularly imprinted cryogel beads for cholesterol removal from milk samples Colloids and Surfaces B: Biointerfaces 2020 190 110860 10.1016/j.colsurfb.2020.110860 32126357

[45] Salehi E Afshar S Mehrizi MZ Chehrei A Asadi M Direct reduction of blood serum cholesterol using Thymus vulgaris L.: Preliminary biosorption study, Process Biochemistry 2018 67 155 164 10.1016/j.procbio.2018.01.023

[46] Yilmaz E Şenel E Ok S Cholesterol removal by selected metal-organic frameworks as adsorbents Journal of Food Science and Technology 2020 57 1 173 181 10.1007/s13197-019-04045-5 31975720 PMC6952505

[47] Dias HMAM Berbicz F Pedrochi F Baesso ML Matioli G Butter cholesterol removal using different complexation methods with beta-cyclodextrin, and the contribution of photoacoustic spectroscopy to the evaluation of the complex Food Research International 2010 43 4 1104 1110 10.1016/j.foodres.2010.02.002

